# On the genus *Crossaster* (Echinodermata: Asteroidea) and its distribution

**DOI:** 10.1371/journal.pone.0227223

**Published:** 2020-01-07

**Authors:** Halldis Ringvold, Truls Moum

**Affiliations:** 1 Sea Snack Norway, Bergen, Norway; 2 Genomics Division, Faculty of Biosciences and Aquaculture, Nord University, Bodø, Norway; University of California, UNITED STATES

## Abstract

Several starfish (Echinodermata, Asteroidea) are keystone species of marine ecosystems, but some of the species are difficult to identify using morphological criteria only. The common sunstar, *Crossaster papposus* (Linnaeus, 1767), is a conspicuous species with a wide circumboreal distribution. In 1900, a closely similar species, *C*. *squamatus* (Döderlein, 1900) was described from the NE Atlantic Ocean, but subsequent authors have differed in their views on whether this is a valid taxon or rather an ecotype associated with temperature variations. We assessed the differentiating morphological characters of specimens from Norwegian and Greenland waters identified as *C*. *papposus* and *C*. *squamatus* and compared their distributions in the NE Atlantic as inferred from research cruises. The field data show that *C*. *papposus* is found mainly in temperate and shallow waters, whereas *C*. *squamatus* resides on the shelf-break in colder, mixed water masses. Intraspecific diversity and interspecific genetic differentiation of the two putative species, and their phylogenetic relationships to several *Crossaster* congeners worldwide, were explored using mitochondrial and nuclear DNA sequences. The molecular evidence suggests that *C*. *papposus* is the more diverse and geographically structured taxon, in line with its wide distribution. *C*. *papposus* and *C*. *squamatus* are closely related, yet clearly distinct taxa, while *C*. *papposus* and *C*. *multispinus* H.L. Clark, 1916, the latter from the South Pacific Ocean, are closely related, possibly sister taxa.

## Introduction

Many starfish (Asteroidea) play important ecosystem roles as top predators, with some acting as keystone species, capable of structuring the communities in which they occur [1–4]. The common sun star *Crossaster papposus* (Linnaeus, 1767) is a typical representative, which belongs to the family Solasteridae and has a wide circumboreal distribution [5]. Within the *Crossaster* genus a total of ten species and four subspecies (including one *nomen nudum*) are currently accepted by the World Register of Marine Species [6]. Clusius [7] made one of the earliest records of *Crossaster*, which he described as “*Stella tredecim radiorum*”, later synonymized with *Crossaster papposus*. Historically, the generic designation of this species and some of its allies has alternated between *Crossaster* and *Solaster* (family Solasteridae). The Solasteridae family appears in the fossil record during the Lower Jurassic and with fairly clear generic characters today, according to Blake [8]. Despite this, there have been several disagreements concerning the genera *Solaster* and *Crossaster*. Agassiz proposed two genera: *Solaster* Forbes, 1839 and *Crossaster* [9] (a genus already erected by Müller and Troschel in 1840), for the two species *Solaster endeca* (Linnaeus, 1771) and *Solaster papposus* (later *Crossaster papposus*) [10]. A number of researchers disagreed, e.g. Viguier, Danielssen and Koren [10], Fisher (in [5], and Mortensen [11]. E.g. Fisher considered *Crossaster* a junior synonym of *Solaster*, despite the different character of the marginals, the abactinal skeleton and spinelets. Today, both genera are accepted by Clark and Downey [5] and international expert groups [6].

*Crossaster papposus*, being a common and widely distributed species in the North Atlantic, was recognised by Carl von Linné at an early point in history. Much later, in 1900, Döderlein described a variety that differed slightly from *C*. *papposus*, and he tentatively termed it *Solaster papposus* var. *squamata* (later *Crossaster squamatus* (Döderlein, 1900) [12]). However, researchers have been unable to reach a consensus on whether *C*. *squamatus* should be considered a valid taxon or rather a morphotype of *C*. *papposus* [e.g. 13, 14]. So far, discrimination between the *papposus* and *squamatus* varieties has been based on morphological characteristics only, which could be strongly influenced by the organism’s environmental and ecological contexts. An integrated approach, considering both morphological and molecular evidence, can refine estimates of differentiation and potentially resolve taxonomic disagreements. Previously, allozyme analysis was successfully used on asteroids to separate species groups within the *Henricia* genus [15]. Resolution is further improved by DNA sequence analysis, and the so-called DNA barcoding gene (the mitochondrial cytochrome oxidase subunit I gene; *COI*) provides a convenient target across the animal kingdom due to the simple inheritance pattern of mitochondrial DNA, and the comprehensive data available for comparison [16]. DNA sequence analyses of *COI* were used to study starfish phylogeny [17], and Ward et al. [18] were able to distinguish 187 of 191 echinoderm species by their *COI*-based barcodes. Mitochondrial markers could be biased, however, due to introgression, lineage sorting, and selective sweeps. Thus, phylogenetic relationships are more reliably recovered by inclusion of nuclear encoded markers.

In the present study, we assessed potentially differentiating morphological characters between *C*. *squamatus* and *C*. *papposus*, based on specimens collected in the North Atlantic Ocean. The distributional patterns of the two types of *Crossaster* in the North Atlantic are discussed in relation to temperature and other environmental parameters. The genetic diversity of the two putative species, and the genetic differentiation between them, were evaluated based on specimens from across the Atlantic, using mitochondrial and nuclear ribosomal DNA (rDNA) sequences. We analysed samples from an additional four congeneric species collected worldwide, to allow for a more representative phylogenetic reconstruction of *Crossaster* relationships.

## Materials and methods

### Specimens and distributional data

**Fig 1:**
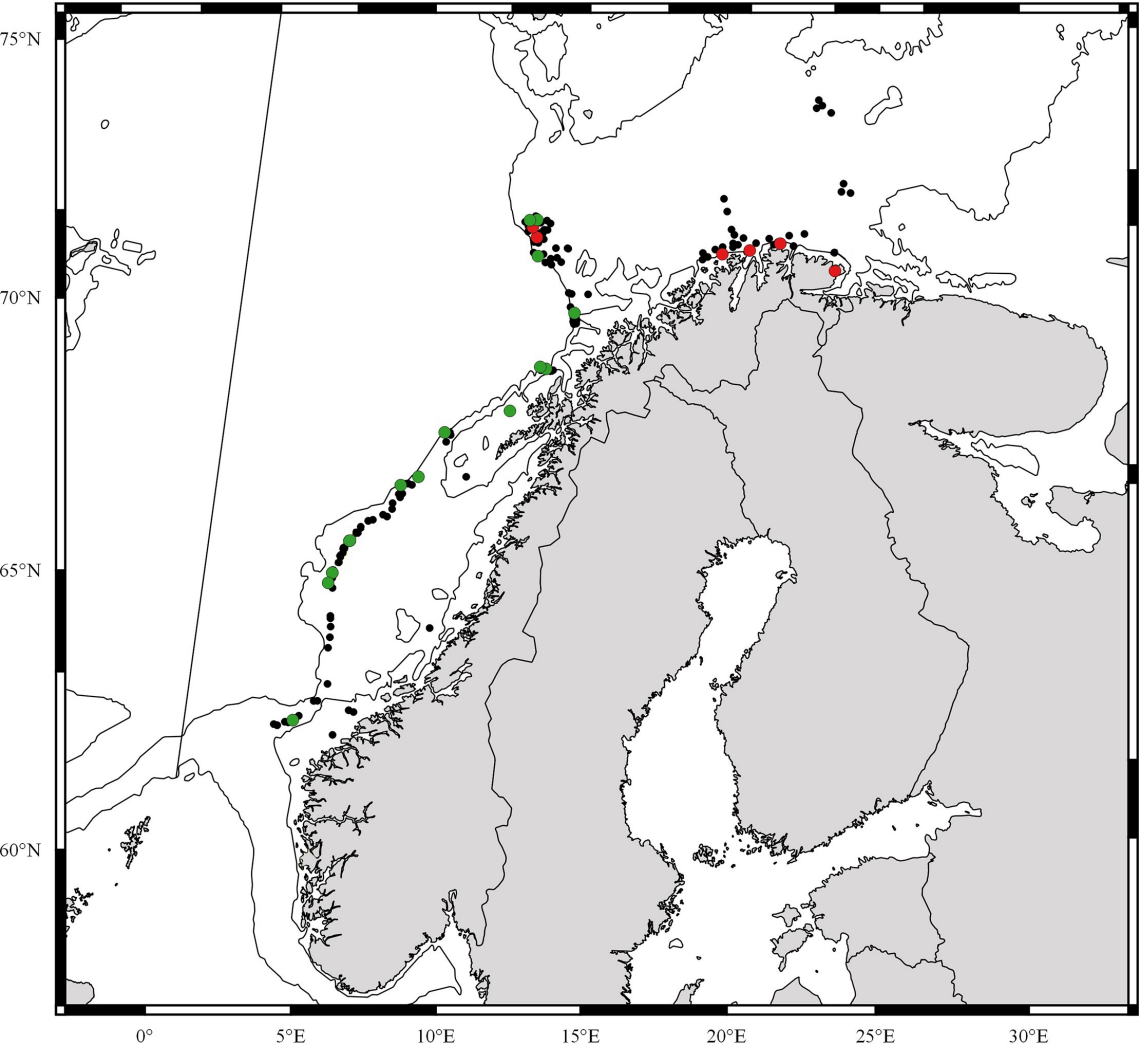
**Sampling stations in the Norwegian Sea where *Crossaster papposus* (red dots) and *C*. *squamatus* (green dots) specimens were recorded, and MAREANO video recordings of *Crossaster* spp. (black dots).** Specimens for DNA analysis were also collected from NW Greenland in the NW Atlantic, and North and South Pacific Ocean. Fig1 is constructed with Quantum GIS (version 12.2.3).

Materials for the present study were mainly collected under the auspices of the ongoing Marine area database for Norwegian waters (MAREANO) program (www.mareano.no) in the NE Atlantic Ocean. The MAREANO program conducts physical, biological, and environmental mapping along the Norwegian coast, based on biannual research cruises. Introduction to, and results from, the 10 first years of the program are given in Buhl-Mortensen et al. [19], and detailed methodologies in Ringvold et al. [20]. While echinoderms collected by MAREANO are normally stored in formalin, ethanol preserved samples for DNA analyses were provided for the present study. In addition, samples were collected during the Ecosystem survey (Institute of Marine Research) in 2016, using methods described in Jørgensen et al. [21], and the Greenland Initiating North Atlantic Benthos Monitoring (INAMon) program, from Melville Bay, NW Greenland in the NW Atlantic Ocean. Three *Crossaster* specimens were collected by HR, scuba diving in shallow waters at Gravdal, Tælavåg and Tellnes, near the city of Bergen, in 2016 (Fig 1). Close-up pictures of the asteroids' dorsal structure were taken with Andonstar 2MP USB Digital Microscope. The specimens are deposited in the collections of the University Museum of Bergen, Norway. The California Academy of Sciences (CAS), USA, and the National Institute of Water and Atmospheric Research Ltd (NIWA), New Zealand, provided ethanol preserved tissue samples (tube feet tissue or whole specimens) for DNA analysis of *C*. *papposus*, *C*. *borealis* Fisher, 1906, *C*. *penicillatus* Sladen, 1889, *C*. *multispinus*, and *C*. *campbellicus* McKnight, 1973 specimens from the Northern and Southern Pacific Ocean (Table 1).

**Table 1:** Starfish specimens and DNA sequences included in this study, the location and year of sampling, latitude and longitude (DD) (approximate DD for all locations), collectors, and GenBank accession numbers. For abbreviations of donators, see Materials and Methods. (*Pictures provided in Fig 2.).

Location/ station nr.	Cruise/program/institution	Year	Latitude	Longitude	Species	Isolate/Museum storage no.	Accession no, COI	Accession no, rDNA
**Norway**								
Gravdal, Bergen	Private diving	2016	60.39422	5.25775	*C*. *papposus*	Cp-2	KX451847*	
Tælavåg, Sotra	Private diving	2016	60.003	4.02389	*C*. *papposus*	Cp-3	MK270376	
Tellnes kai, Sotra	Private diving	2016	60.00167	5.01667	*C*. *papposus*	Cp-4		MK203712*
110, 1218–471	IMR/ MAREANO	2013	70.59306	30.95056	*C*. *papposus*	Cp-1	KX451846	
205, 1086–438	IMR/ MAREANO	2013	66.28544	6.28391	*C*. *squamatus*	Cs-8	MK270383	MK203719
205, 1086–438	IMR/ MAREANO	2013	66.28544	6.28391	*C*. *squamatus*	Cs-9	KX451843	MK203720
205, 1086–438	IMR/ MAREANO	2013	66.28544	6.28391	*C*. *squamatus*	Cs-10	KX451844	MK203721
205, 1086–438	IMR/ MAREANO	2013	66.28544	6.28391	*C*. *squamatus*	Cs-11	KX451845	MK203722
205, 1093–439	IMR/ MAREANO	2013	65.95742	5.84827	*C*. *squamatus*	Cs-3	KX451838	MK203715
205, 1093–439	IMR/ MAREANO	2013	65.95742	5.84827	*C*. *squamatus*	Cs-4	KX451839	MK203716
205, 1093–439	IMR/ MAREANO	2013	65.95742	5.84827	*C*. *squamatus*	Cs-5	KX451840	MK203717
205, 1093–439	IMR/ MAREANO	2013	65.95742	5.84827	*C*. *squamatus*	Cs-6	KX451841	
205, 1093–439	IMR/ MAREANO	2013	65.95742	5.84827	*C*. *squamatus*	Cs-7	KX451842	MK203718
693	IMR/ Eco cruise	2016	72.7985	22.67562	*C*. *papposus*	Cp-5	MK270377	MK203713
699	IMR/ Eco cruise	2016	74.2954	17.46801	*C*. *papposus*	Cp-6	MK270378	MK203714
737	IMR/ Eco cruise	2016	73.1951	20.07648	*C*. *squamatus*	737–1	MK270379	MK203723
737	IMR/ Eco cruise	2016	73.1951	20.07648	*C*. *squamatus*	737–2	MK270380	MK203724
737	IMR/ Eco cruise	2016	73.1951	20.07648	*C*. *squamatus*	737–3	MK270381	MK203725
737	IMR/ Eco cruise	2016	73.1951	20.07648	*C*. *squamatus*	737–4	MK270382*	MK203726
**Greenland**								
PA-7-50	INAMon	2016	74.94193	-62.59835	cf. *C*. *papposus*	PA-7-50		MK203727
PA-7-56	INAMon	2016	75.10695	-62.63746	*C*. *papposus*	PA-7-56A	MK270384	
PA-7-67	INAMon	2016	75.49691	-63.57091	*C*. *papposus*	PA-7-67	MK270385	MK203728
PA-7-69	INAMon	2016	75.47533	-64.08251	*C*. *papposus*	PA-7-69C	MK270386	MK203729
PA-7-108	INAMon	2016	73.83896	-58.73608	*C*. *papposus*	PA-7-108B	MK270387	PA-7-108A
PA-7-108	INAMon	2016	73.83896	-58.73608	*C*. *papposus*	PA-7-108A		MK203730
PA-7-119	INAMon	2016	73.48046	-59.28395	*C*. *papposus*	PA-7-119H	MK270388	MK203731
PA-7-119	INAMon	2016	73.48046	-59.28395	*C*. *papposus*	PA-7-119J	MK270389	MK203732
PA-7-120	INAMon	2016	73.48378	-59.28004	*C*. *papposus*	PA-7-120A	MK270390	MK203733
PA-7-123	INAMon	2016	73.51105	-60.4544	*C*. *papposus*	PA-7-123	MK270391	
**USA (incl. Alaska), Arctic, Antarctica, Pacific**								* *
USA, California, Fort Bragg	CAS	1998	39.01722	-124.055	*C*. *borealis*	CASIZ 115077-A		MK203734
USA, Alaska	CAS	1999	58.50777	-140.18055	*C*. *papposus*	CASIZ 171926		MK203735
Arctic Ocean, outer continental shelf	CAS	1977	70.16666	-141.00167	*C*. *papposus*	CASIZ 163273		MK203736
Antarctica, South Orkney Islands	CAS	2009	-60.8702	-43.1962	*C*. *penicillatus*	CASIZ 180771	MK270392	
Antarctica, South Orkney Islands	CAS	2009	-60.763	-43.4223	*C*. *penicillatus*	CASIZ 180782		MK203737
**New Zealand**								
New Zealand, South	NIWA	2012	-49.05	166.58333	*C*. *campbellicus*	75833/ TRIP3440/6	MK270393*	MK203738
New Zealand, East	NIWA	2006	-42.7575	179.9922	*C*. *multispinus*	27308/ TAN0604/15		MK203739
New Zealand, South	NIWA	2007	-49.31433	166.62683	*C*. *multispinus*	46150/ TAN0714/91	MK270394	
**GenBank samples**								
Canada, British Columbia					*C*. *papposus*		HM542135	
Canada, British Columbia					*C*. *papposus*		HM473903	* *
Canada, British Columbia					*C*. *papposus*		HM542132	
Canada, British Columbia					*C*. *papposus*		HM542133	
Canada, British Columbia					*C*. *papposus*		HM542131	
Canada, British Columbia					*C*. *papposus*		HM473902	
Canada, British Columbia					*C*. *papposus*		HM542134	
USA, East Pacific, Washington					*C*. *papposus*		AF217383	
Canadian Arctic, Nunavut					*C*. *papposus*		HM543003	
Canadian Arctic, Nunavut					*C*. *papposus*		HM543002	
Canada, British Columbia, W of Cape St. James					*C*. *borealis*		HM542925	
Canada, British Columbia, W of Cape St. James					*Heterozonias alternatus*		HM542931	
Canada, British Columbia, Barkley sound					*Lophaster furcilliger*		HM542934	
Canada, Nunavut, Resolute Bay					*Solaster* sp.		HM543069	
Canada, BC, Nanaimo, Departure Bay					*Solaster* sp.		HM542376	
Canada, BC, Nanaimo, Departure Bay					*S*. *dawsoni*		HM542365	
Canada, St. Andrews, Spruce Island					*S*. *endeca*		HM542371	
Canada, BC, West of Cape Scott					*S*. *paxillatus*		HM542990	
USA, Washington, San Juan Islands					*S*. *stimpsoni*		AF217382	

**Fig 2:**
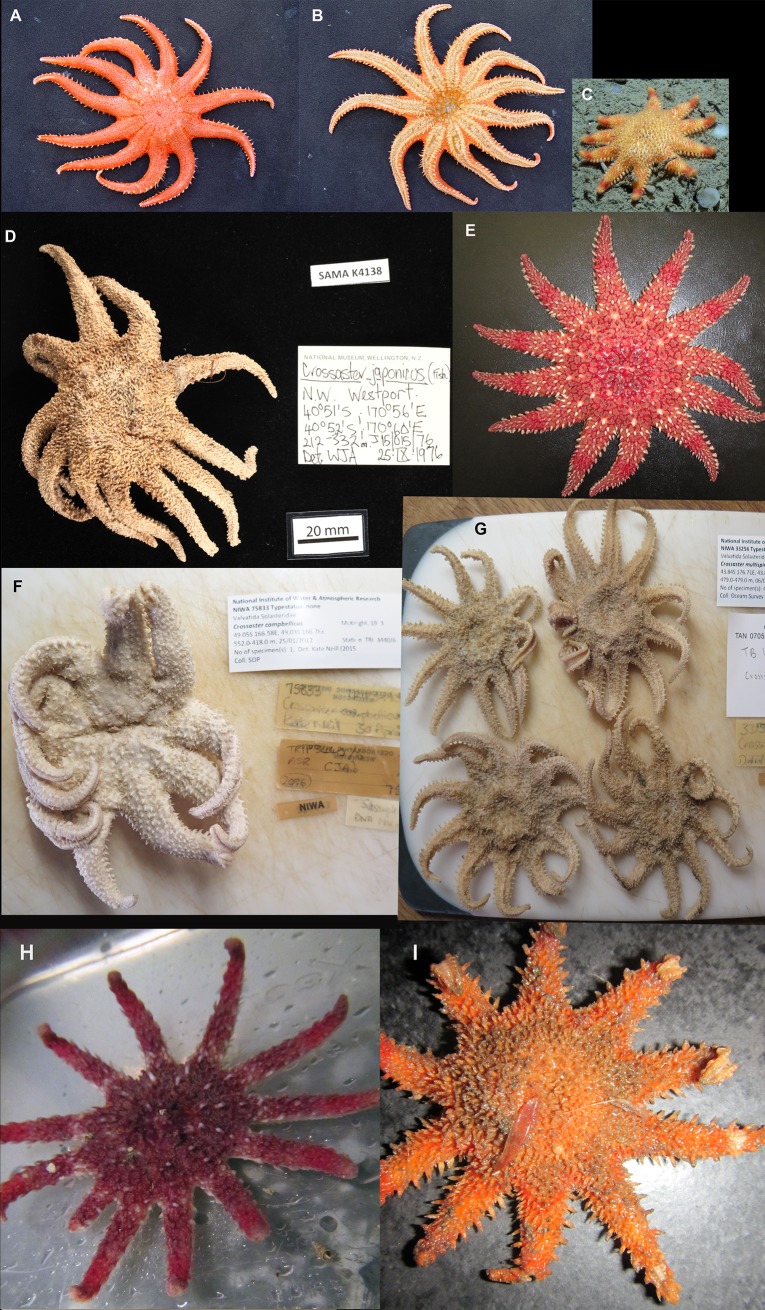
Photographic representations of *Crossaster* worldwide. 2a. *C*. *borealis* (dorsal side) from Alaska, Bering Sea. 2b. *C*. *borealis* (ventral side, same specimen as 2a). 2c. Live *C*. *squamatus* from West of Shetland, September 2009, at 1050 m depth. Identified from video image by Daniel Jones. 2d. *C*. *japonicus* (Fisher, 1911) from NW Westport, New Zealand. 2e. Live *C*. *papposus* from Gravdal, near the city of Bergen, Norway. 2f. Ethanol preserved *C*. *campbellicus* from South New Zealand. 2g. Ethanol preserved *C*. *multispinus* from East New Zealand. 2h. Live *C*. *papposus* from Tellnes, near Bergen, Norway. 2i. Frozen *C*. *squamatus* from Barents Sea, IMR/ Ecocruise, st. 737. (Photo credits: 2a and 2b by Roger Clark, 2c by Daniel Jones/ SERPENT Project, National Oceanography Centre, 2d by Geoff Lemmey, CC license/ South Australian Museum and 2e-2i by Halldis Ringvold/ Sea Snack Norway.).

* Pictures are provided in Fig 2.

Data on locations, depth and temperature were available for the *Crossaster* samplings mentioned above. In addition, we made use of the corresponding data associated with *Crossaster* specimens collected by the Marine benthic fauna of the Faroe Islands program (BIOFAR) and the Benthic Invertebrates of Icelandic Waters program (BIOICE). Information on sampling methods for these cruises is given in Ringvold and Andersen [22], Dauvin et al. [23] and Ringvold et al. (In prep.). Identification was based on Mortensen [11] and Clark and Downey [5].

### DNA sequence analyses

Genomic DNA was extracted from tube feet of ethanol-preserved specimens using the DNeasy Blood & Tissue Kit (QIAGEN) according to the manufacturer’s instructions. An 841 bp fragment from the 5’ end of *COI* was PCR amplified using primers EchinoF1 [18] and COIer [24]. A fragment of the rDNA array was amplified using echinoderm targeting primers 18d9 and 5.8Sr, described by Petrov et al. [25], which we subsequently redesigned for increased specificity towards *Crossaster*: 18Scro1f (GTAGGTGAACCTGCGGAAGGATC) and 5.8Scro1rev (ATGTCGATGATCACTGCGTTCTGC). The resulting ~ 500 bp PCR product contains the variable internal transcribed spacer 1 (ITS1) of approximately 390 bp, and short flanking rDNA sequences (partial 18S, ~20 bp; partial 5.8S, ~100 bp); gene borders inferred from sequence comparisons to *Asterias amurensis* Lutken, 1871 (GenBank KX592567). PCR was performed in 20 μl volumes using the AmpliTaq Gold 360 system, containing 0.25 μM of each primer and 2.5 mM MgCl_2_. Cycling parameters for the amplification reactions were 95°C for 3 min, followed by 35 cycles of denaturation at 95°C for 0.5 min; annealing at 50°C (*COI*) or 62°C (rDNA) for 1 min; extension at 72°C for 1 min, and a final elongation step at 72°C for 10 min. Amplification products were sequenced on both strands using the BigDye v3.1 kit and Applied Biosystems 3500xL Genetic Analyzer.

Given the history of shifting taxonomic designations among *Crossaster* and *Solaster* species, we compiled available *COI* sequences (≥ 841 nucleotides) assigned to the two genera, as well as *Heterozonias alternatus* [originally *Crossaster alternatus* (Fisher, 1906)], for phylogenetic analysis. Phylogenetic relationships among the species were inferred using a representative *COI* sequence from each taxon and *Lophaster furcilliger* Fisher, 1905 (Solasteridae) for outgroup rooting. The resulting phylogeny confidently grouped *C*. *papposus*, *C*. *multispinus* and *C*. *squamatus* to the exclusion of other *Crossaster* and *Solaster* species. Thus, the phylogenetic relationships within and among *C*. *papposus*, *C*. *multispinus* and *C*. *squamatus* were further analysed using *COI* sequences from all specimens, and *C*. *borealis* for outgroup rooting.

Sequences were aligned using Muscle [26] with default parameters as implemented in MEGA X version 10.0.5 [27], and MEGA was further used to recover phylogenetic relationships based on mitochondrial and nuclear DNA sequences by the Maximum Likelihood method. The most appropriate model of sequence substitution for each phylogenetic analysis was determined based on the lowest Bayesian Information Criterion (BIC) score among 24 alternative models. For the interspecific phylogenetic representation based on *COI*, the GTR (General Time Reversible) model was selected, with 58% invariable sites and non-uniformity of evolutionary rates among variable sites modelled using a discrete gamma distribution with 5 rate categories and a gamma parameter of 0.9279. Phylogenetic trees incorporating inter-individual relationships for *C*. *papposus* and *C*. *squamatus*, were recovered separately based on *COI* and rDNA data. The Tamura 3-parameter model was selected for both data sets, using a gamma distribution with 5 rate categories and gamma parameters of 0.0933 and 0.1435, respectively. For each phylogenetic tree, bootstrap support values were calculated using 1,000 replicates. Intraspecific nucleotide diversities and the number of nucleotide substitutions per site between *C*. *papposus* and *C*. *squamatus* were estimated in DnaSP version 6.12.01 [28] using the Jukes-Cantor model.

## Results

### Morphology

Among 26 ethanol conserved *Crossaster* specimens from Norwegian and Greenland waters that were chosen for morphological analysis, 6 and 20 were identified as *C*. *papposus* and *C*. *squamatus*, respectively (Tables 1 and 2). A total of 6 *C*. *multispinus* was received from New Zealand. Representative *Crossaster* species from around the world are shown in Fig 2.

**Table 2:** Starfish specimens included in this study, depth (m), temperature (°C), species, amount and major radii (R, cm).

Location/ station nr.	Depth	Temp.	Species	Amount	R
**Norway**					
Gravdal, Bergen	~10	**-**	*C*. *papposus*	1	13,0
Tælavåg, Sotra	~10	**-**	*C*. *papposus*	1	9,0
Tellnes kai, Sotra	~10	12	*C*. *papposus*	1	3,3
110, 1218–471	157	5,38	*C*. *papposus*	1	2,0
205, 1086–438	565	2,35	*C*. *squamatus*	4	0,6/ 0,7/ 1,7/ 1,8
205, 1093–439	608	0,77	*C*. *squamatus*	5	0,9/ 1,1/ 1,2/ 1,3/ 2,2
103, 1723–11	318	4,2	*C*. *squamatus*	7	na
693	400	4,18	*C*. *papposus*	1	2,5
699	172	5,22	*C*. *papposus*	1	1,8
737	445	3,31	*C*. *squamatus*	4	1,3/ 1,8/ 2,7/ 3,2
*Norway*			* *	*26*	
**Greenland**					
PA-7-50	358	2.32	cf. *C*. *papposus*	1	1,1
PA-7-56	176	1.18	*C*. *papposus*	11	1,8/ 1,8/ 2,0/ 2,0/ 3,0 +6 ind.<1,5
PA-7-67	185	1.72	*C*. *papposus*	1	2,0
PA-7-69	175	1.31	*C*. *papposus*	4	1,0/ 1,3/ 1,5/ 2,0
PA-7-108	159	0.71	*C*. *papposus*	2	0,9/ 3,2
PA-7-119	180	0.91	*C*. *papposus*	25	1,6/ 1,7/ 1,7 + 22 ind< 1.5
PA-7-120	180	1	*C*. *papposus*	2	1,3/ 2,1
PA-7-123	355	2.32	*C*. *papposus*	1	1,8
*Greenland sum*	* *	* *	* *	*47*	* *
**USA (incl. Alaska), Arctic, Antarctica, Japan, and Philippines**					
USA, California, Fort Bragg	530		*C*. *borealis*	1	
USA, Alaska, Aleutian Islands	400		*C*. *borealis*	1	
USA, Alaska, Bering Sea	876		*C*. *borealis*	1	
USA, Alaska, Bering Sea	627		*C*. *borealis*	1	
USA, shelf off Oregon	1187		*C*. *borealis*	1	
USA, Alaska, Island of four Mountains	102		*C*. *papposus*	1	
USA, Alaska, Aleutian Islands	83		*C*. *papposus*	1	
USA, Alaska	343		*C*. *papposus*	1	
USA, Alaska, Bering Sea	1090		*C*. *papposus*	1	
USA, Alaska, Bering Sea	548		*C*. *papposus*	1	
Arctic Ocean, outer continental shelf	50		*C*. *papposus*	1	
Japan, East China Sea, W of Kyushu	80		*C*. *papposus*	1	
Japan, Toshima Island	?		*C*. *papposus*	1	
Antarctica, South Orkney Islands	~340		*C*. *penicillatus*	1	
Antarctica, South Orkney Islands	300		*C*. *penicillatus*	1	
Philippines, Luzon Island	?		*C*. *scotophilus*	1	
*USA (incl*. *Alaska)*, *Arctic*, *Antarctica*, *Japan*, *and Philippines sum*				*16*	
**New Zealand**					
New Zealand, East	340		*C*. *campbellicus*	1	** **
New Zealand, South	?		*C*. *campbellicus*	1	** **
New Zealand, South	418–552		*C*. *campbellicus*	1	** **
New Zealand, East	830–1060		*C*. *multispinus*	1	** **
New Zealand, East	479		*C*. *multispinus*	4	** **
New Zealand, South	663–673		*C*. *multispinus*	1	** **
*New Zealand sum*	* *	* *	* *	*9*	* *

Class Asteroidea de Blainville, 1830

Superorder Valvatacea Blake, 1987

Order Valvatida Perrier, 1884

Family Solasteridae Viguier, 1878

***Crossaster*** Müller & Troschel, 1840

**Fig 3:**
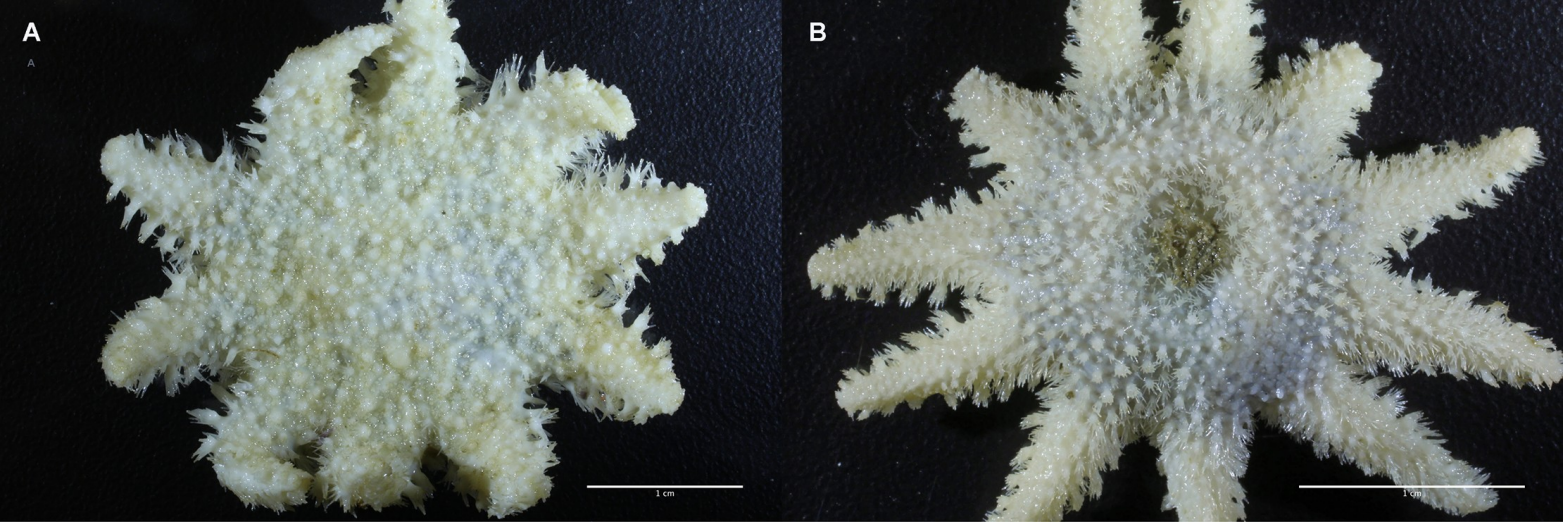
a (left). Whole, conserved specimens of small *Crossaster papposus*, and b. *C*. *squamatus*. They are recorded from MAREANO stations 1218–471 (R = 2 cm), and 1086–438 (R = 1,8 cm), respectively. (Photo credit: Arne Hassel/ Institute of Marine Research.).

**Fig 4:**
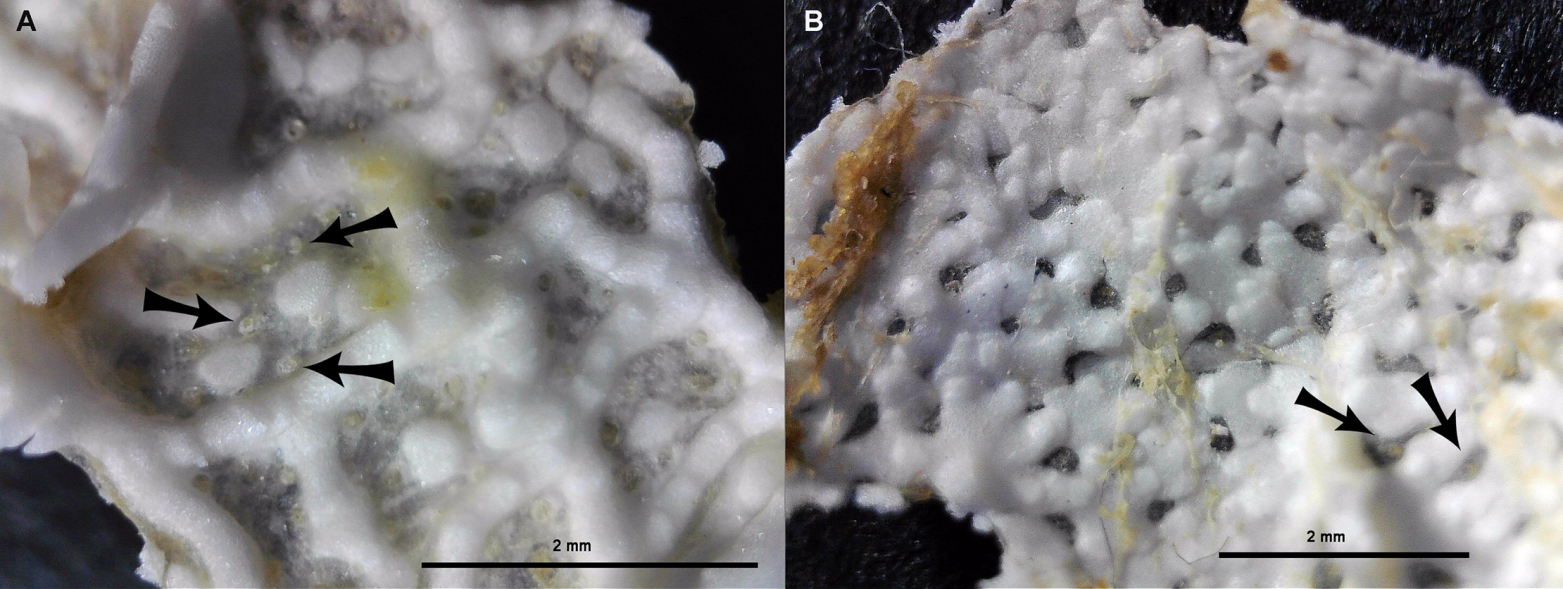
The dorsal skeleton of *Crossaster papposus* is formed by narrow bars with large membranaceous spaces. Specimen recorded at MAREANO station 1218–471 (R = 2 cm). The dorsal skeleton sample is cut out and photographed from below. The arrows show papulae within membranaceous space. (Photo credit: Halldis Ringvold/ Sea Snack Norway.).

*Crossaster* is a genus of Solasteridae with 8–15 tapering arms, moderate to large disc, and single series of single conspicuous marginals visible from dorsal view [5]. *Crossaster papposus* (Fig 3A) and *C*. *squamatus* (Fig 3B) differ morphologically in several structures. In *C*. *papposus*, the dorsal skeleton consists of narrow bars forming an irregular reticulum of plates [11]. All our purported *C*. *papposus* exhibited this structure (Fig 4). Large membranaceous spaces are formed within the reticulum, and in these spaces, several papulae can be found. The dorsal paxillae are unequal in size [29], and marginal paxillae largest. According to Mortensen [11] there are 3–5 furrow spines, which is in agreement with our specimens. Specimens of *Crossaster* are variable in colouration, however, the predominant aboral colour of *C*. *papposus* is purple-red, arms sometimes having a whitish and/or dark red band(s), and the oral side is usually white, which is the case for our live specimens (Fig 2E and 2H).

**Fig 5:**
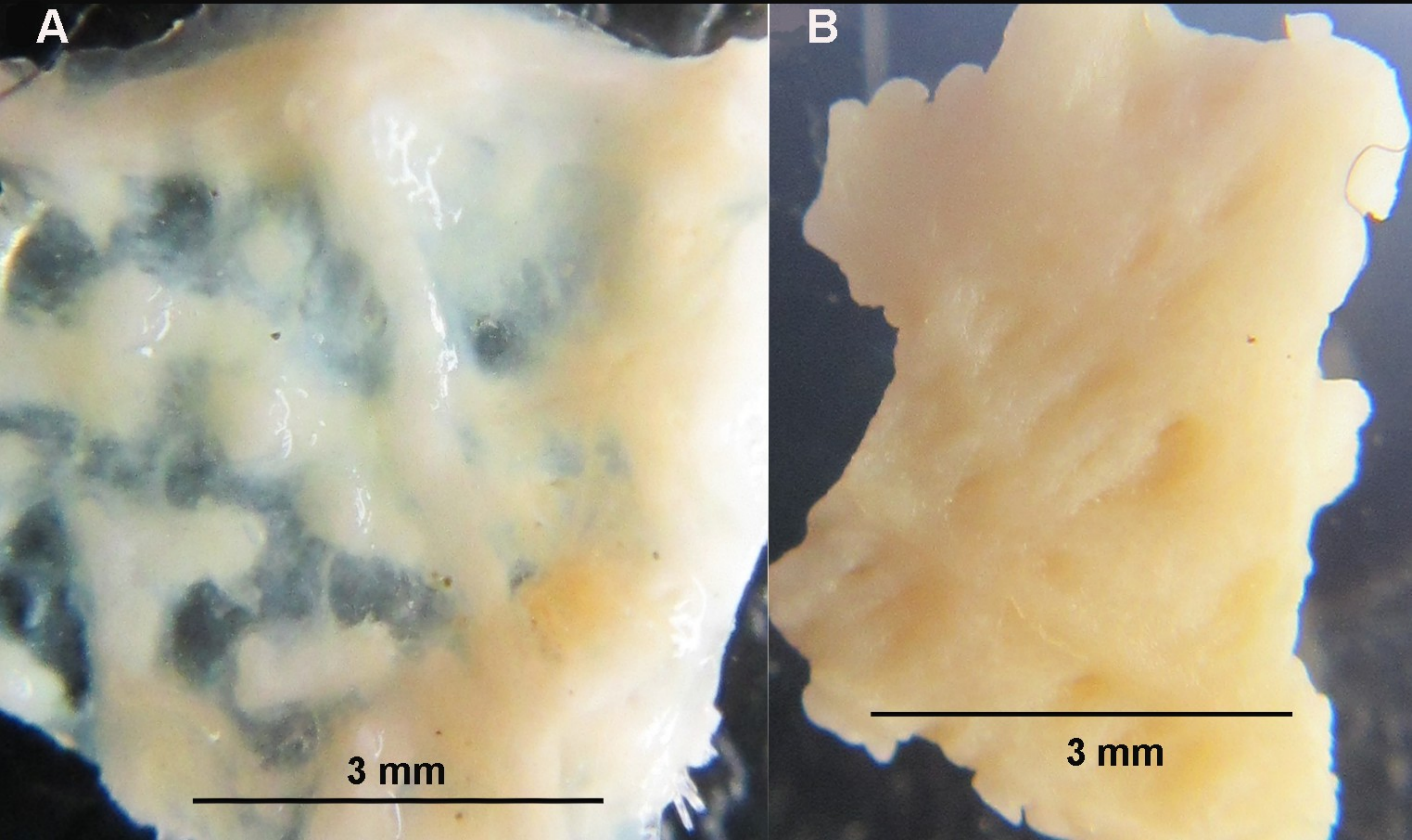
The dorsal skeleton of *Crossaster squamatus* is scale-like, with irregular shaped plates, and with little membranaceous space. Specimen recorded at MAREANO station 1086–438 (R = 1,8 cm). The dorsal skeleton sample is cut out and photographed from below. The arrows show papulae within membranaceous space. (Photo credit: Halldis Ringvold/ Sea Snack Norway.).

In *C*. *squamatus*, the dorsal skeleton is scale-like, formed by irregularly shaped plates with little or no membranaceous spaces, as seen in the MAREANO specimens (Fig 5), and with only singular papula. The aboral paxillae are equal in size, and shorter than for *C*. *papposus* [29]. There are 5–7 furrow spines. The aboral color is usually orange-red, and arms sometimes with orange or red colored bands (Fig 2C and 2I), and the oral side yellowish-white [11].

The dorsal skeleton of the borrowed *C*. *multispinus* consists of narrow bars forming an irregular reticulum of plates with large membranaceous spaces, as in *C*. *papposus*, and also described by Clark [30]. However, the papulae are few and isolated. Marginal paxillae of *C*. *multispinus* are largest, and dorsal paxillae are unequal in size, as for *C*. *papposus*. *C*. *multispinus* has 8–9 adambulacral spines and 10 furrow spines, whereas *C*. *papposus* has 6–7 adambulacral spines and 3–5 furrow spines. According to Clark [30] *C*. *multispinus* has 11 arms with R = 4 cm and r = 2 cm, whereas *C*. *papposus*, according to Clark & Downey [5], is a larger species with 11–14 arms and R = 5,5 cm and r = 3 cm.

The most striking feature of *C*. *papposus* and its closest relatives (*C*.*papposus*/ *C*. *squamatus*/ *C*. *multispinus*), is the shape of the paxillae (Table 3). They all have what could be referred to as high metapaxillae, that is paxillae with high columnar plate. This is in contrast to the phylogenetic cluster containing *Heterozonias alternatus* and associated species, of which species have paxillae with low columnar plate. Paxillae for several of these latter species are in literature named pseudopaxillae, low metapaxillae or small paxillae [e.g. 5, 31, 32].

**Table 3:** Identification key on morphological differences between *Crossaster papposus*, *C*. *squamatus* and *C*. *multispinus*. Color of live specimens vary, but colors in general is given. Comments from this study in italics. Locations of holotypes.

Species	Size (R, in mm)	Dorsal skeleton	Dorsal papulae	Dorsal paxillae	Dorsal paxillar spinelets	Live colour	Furrow spines	Comments	References
*Crossaster multispinus*	40	Open meshwork of narrow ossicles; Abactinal plates variously lobate, centres raised into a distinct pedicel; skeletal meshes relatively large, irregular in outlines	Few, large	High meta-paxillae, unevenly sized; distal paxillae slightly smaller; all paxillae irregularly arranged on abactinal surface and side of arms	Abactinal paxillae have delicate, sharply pointed spinelets; tufts of 8–15 long spines, extending the stalk in length; up to 30 in the larger paxillae	Yellowish-brown (dry specimens)		"Pseudopaxillae", in Clark [30]. In our study only conserved specimens are studied	[30, 33]
*C*. *papposus*	170	Narrow bars forming an open, irregular network with large membranaceous spaces	Multiple papulae in each group	High meta-paxillae; unevenly sized	Long, slender spinelets; 20 or more in each paxillae	Red/ purple	3–5	"High pseudopaxillae", termed used in Verrill (1914)	[5, 11]
*C*. *squamatus*	~53	Scale-like, small spaces between plates	Single papula	High meta-paxillae; paxillae mainly as for the above species, but rather smaller and of more uniform size	Paxillae mainly like for C. papposus, but smaller	Orange/ red	5–7		[11, 12]
**Locations of Crossaster holotypes in our study:**									
*Crossaster borealis*		USA, Kadiak [sic] Island, Alaska							[31]
*C*. *campbellicus*		New Zealand							[34]
*C*. *multispinus*		Australia, Tasmania, Bruny Island							[30]
*C*. *papposus*		Europe or Asia							[5]
*C*. *penicillatus*		Southern Island, between Nightingale Island and Marion Island							[35]
*C*. *scotophilus*		Indonesia, Sulawesi (Celebes), Gulf of Boni							[36]
*C*. *squamatus*		Norway, Eggakanten							[12]
*Heterozonias alternatus*		USA, California, Santa Barbara Islands							[31]
*Lophaster furcilliger*		USA, between Santa Barbara Islands and San Nicholas Islands							[37]

### Distribution

**Fig 6:**
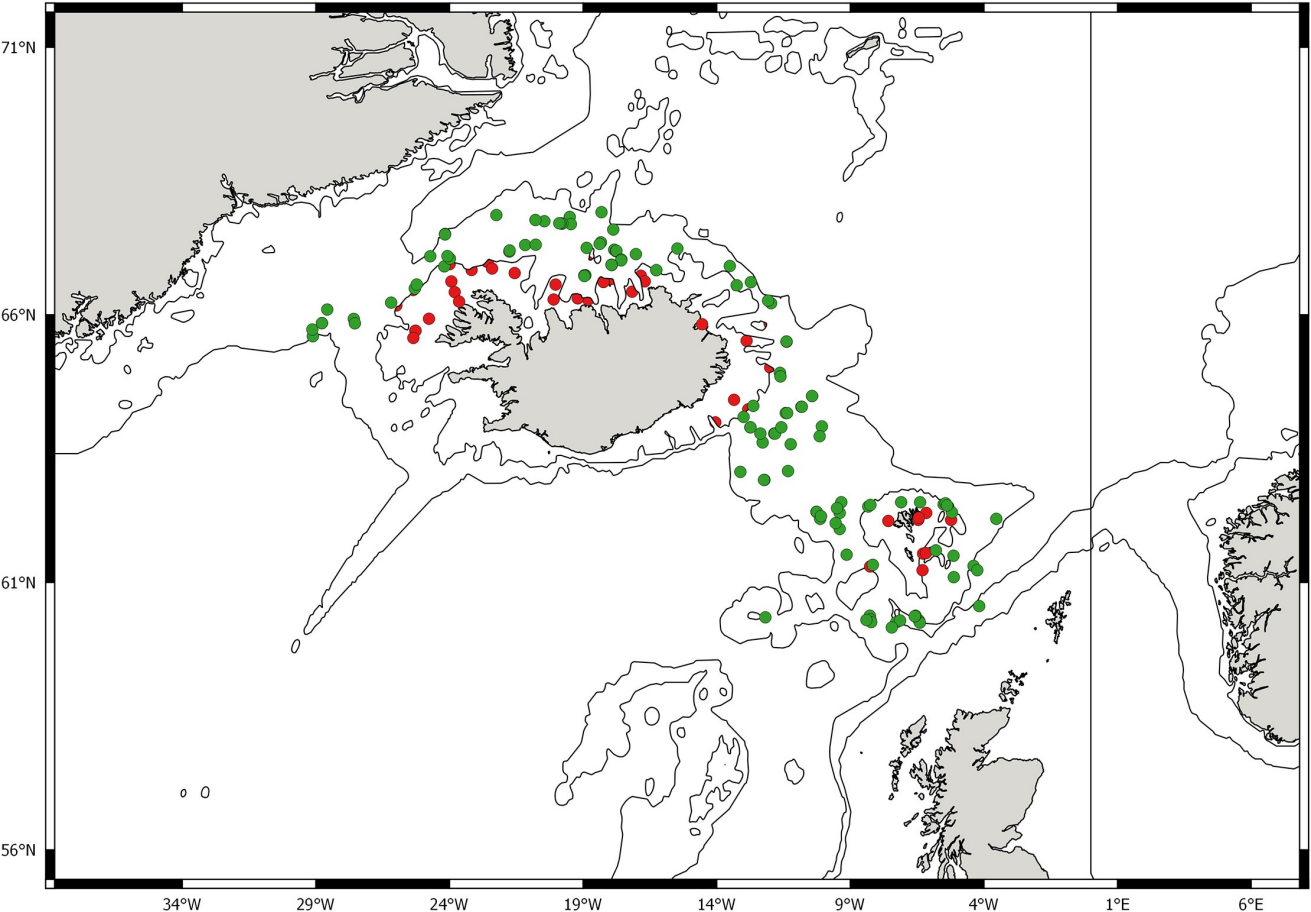
Distribution of *Crossaster papposus* (red dots) and *C*. *squamatus* (green dots) recorded by the BIOFAR and BIOICE programs [22, Ringvold et al. In prep.].

**Fig 7:**
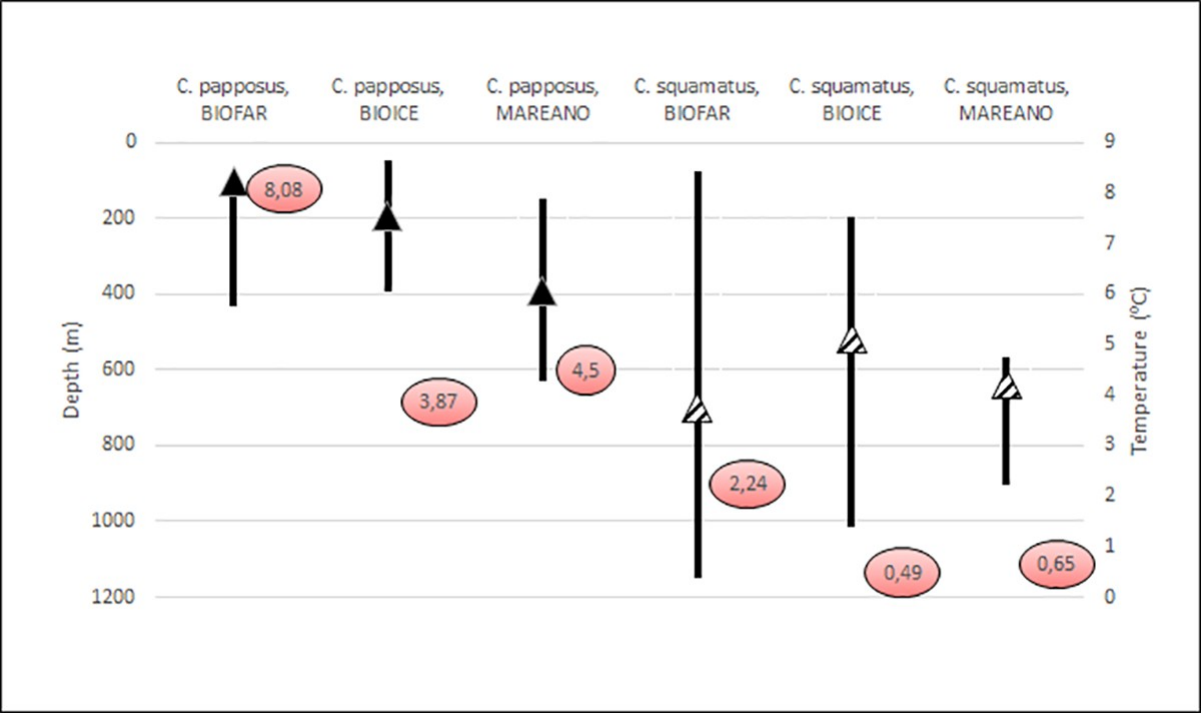
Distribution of *Crossaster papposus* and *C*. *squamatus* from the Faroe Island, Iceland and Norway (data collected by the BIOFAR, BIOICE and MAREANO programs, respectively), in relation to depth and sea floor temperature recorded. The vertical bars indicate the minimum and maximum depths; triangles abundance-weighted mean depth; red circles abundance-weighted mean sea floor temperatures.

Results from the three marine surveillance programs MAREANO, BIOICE and BIOFAR indicate that *C*. *papposus* is mainly recorded from the shelf, in temperate water, whereas *C*. *squamatus* occurs at the shelf-break in colder water (Figs 6 and 7).

### Molecular analyses

**Fig 8:**
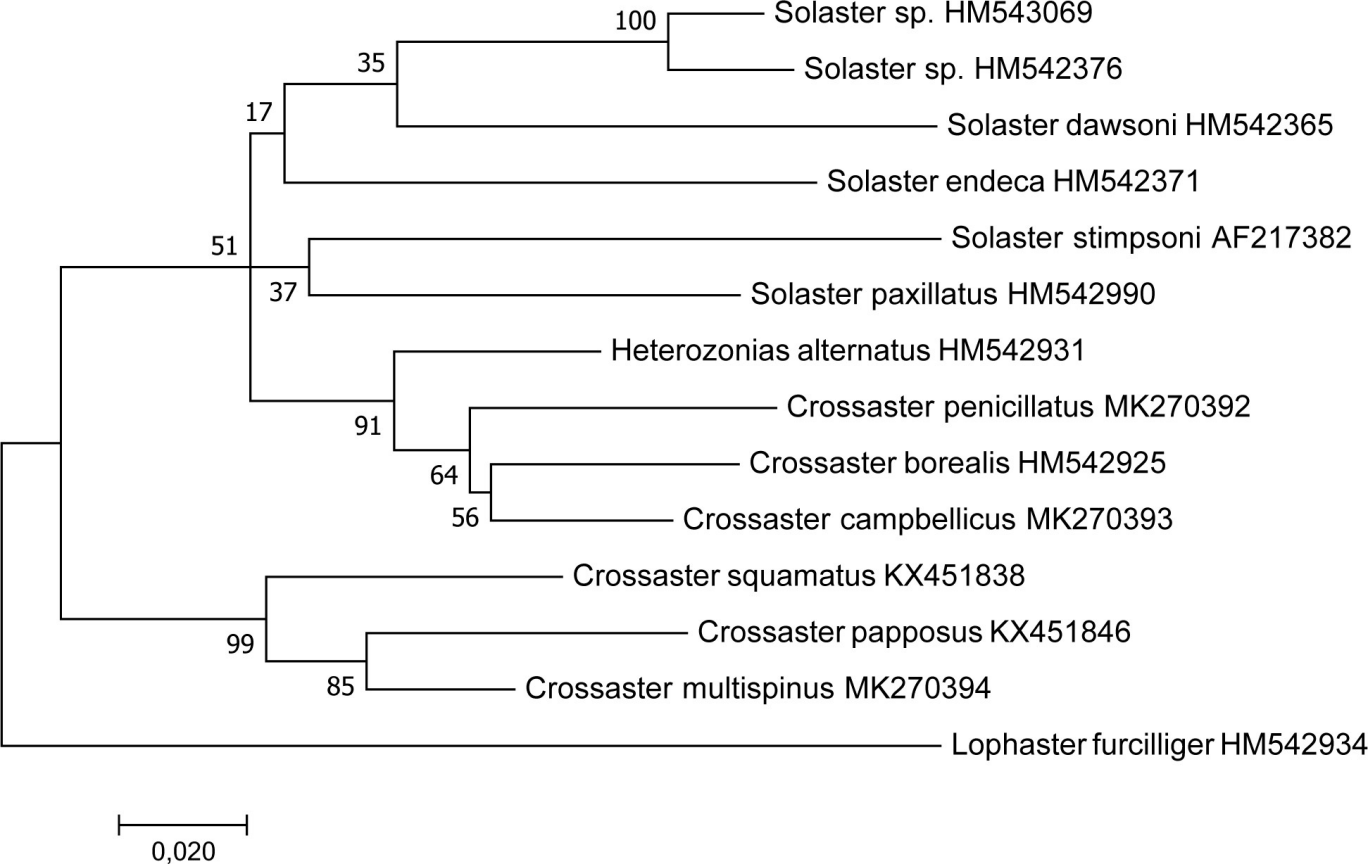
Phylogeny of *Crossaster* and *Solaster* species based on *COI*. The phylogeny was inferred from mitochondrial *COI* sequences using the Maximum Likelihood method and *Lophaster furcilliger* as the outgroup. The tree with the highest log likelihood (-3528,66) is shown. The percentage of bootstrap replicates in which the associated taxa clustered together is shown next to the branches. The tree is drawn to scale, with branch lengths measured by the number of substitutions per site.

Phylogenetic reconstruction of *Crossaster* and *Solaster* species based on *COI* sequences from representative specimens of each species, suggested two clearly defined main groups with high bootstrap support (Fig 8). Firstly, *C*. *squamatus*, *C*. *papposus* and *C*. *multispinus* formed a highly supported clade, and a sister group relationship between *C*. *papposus* and *C*. *multispinus* was supported by a bootstrap value of 85. Secondly, *H*. *alternatus*, *C*. *penicillatus*, *C*. *borealis*, and *C*. *campbellicus* constituted a robust group, while the relationships within this group were less confidently resolved (bootstrap values ≤ 64). The phylogeny of *Solaster* species was not confidently resolved by this analysis due to low bootstrap support values.

**Fig 9:**
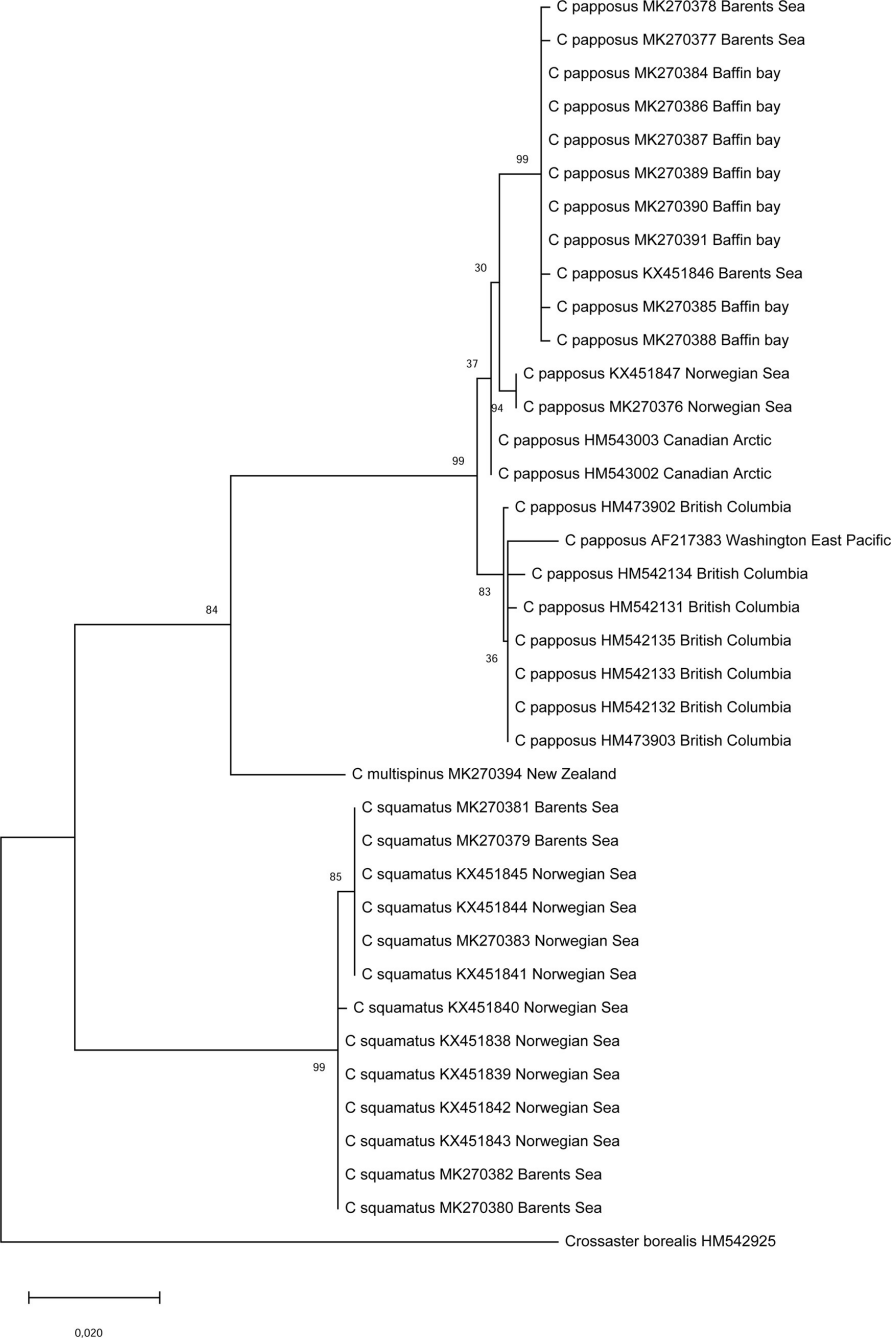
Phylogenetic relationships of the focal taxa based on *COI*. Relationships among *Crossaster papposus* and *C*. *squamatus* individuals, and *C*. *multispinus*, as inferred from mitochondrial *COI* sequences using the Maximum Likelihood method and *C*. *borealis* as the outgroup. The tree with the highest log likelihood (-1972,69) is shown. The percentage of bootstrap replicates in which the associated taxa or individuals clustered together is shown next to the branches. The tree is drawn to scale, with branch lengths measured by the number of substitutions per site.

The relationships between *Crossaster* species were further scrutinized using both mitochondrial *COI* sequences and nuclear rDNA sequences, taking the inter-individual variations of *C*. *squamatus* and *C*. *papposus* into account. The phylogenetic reconstruction based on *COI* and outgroup rooted by *C*. *borealis* recovered *C*. *papposus* and *C*. *squamatus* as clearly separate units with high bootstrap support, and the grouping of *C*. *multispinus* and *C*. *papposus* gained further support by a bootstrap of 84. While *C*. *squamatus* exhibited low genetic differentiation among individuals, *C*. *papposus* showed evidence of phylogeographic structuring (Fig 9).

**Fig 10:**
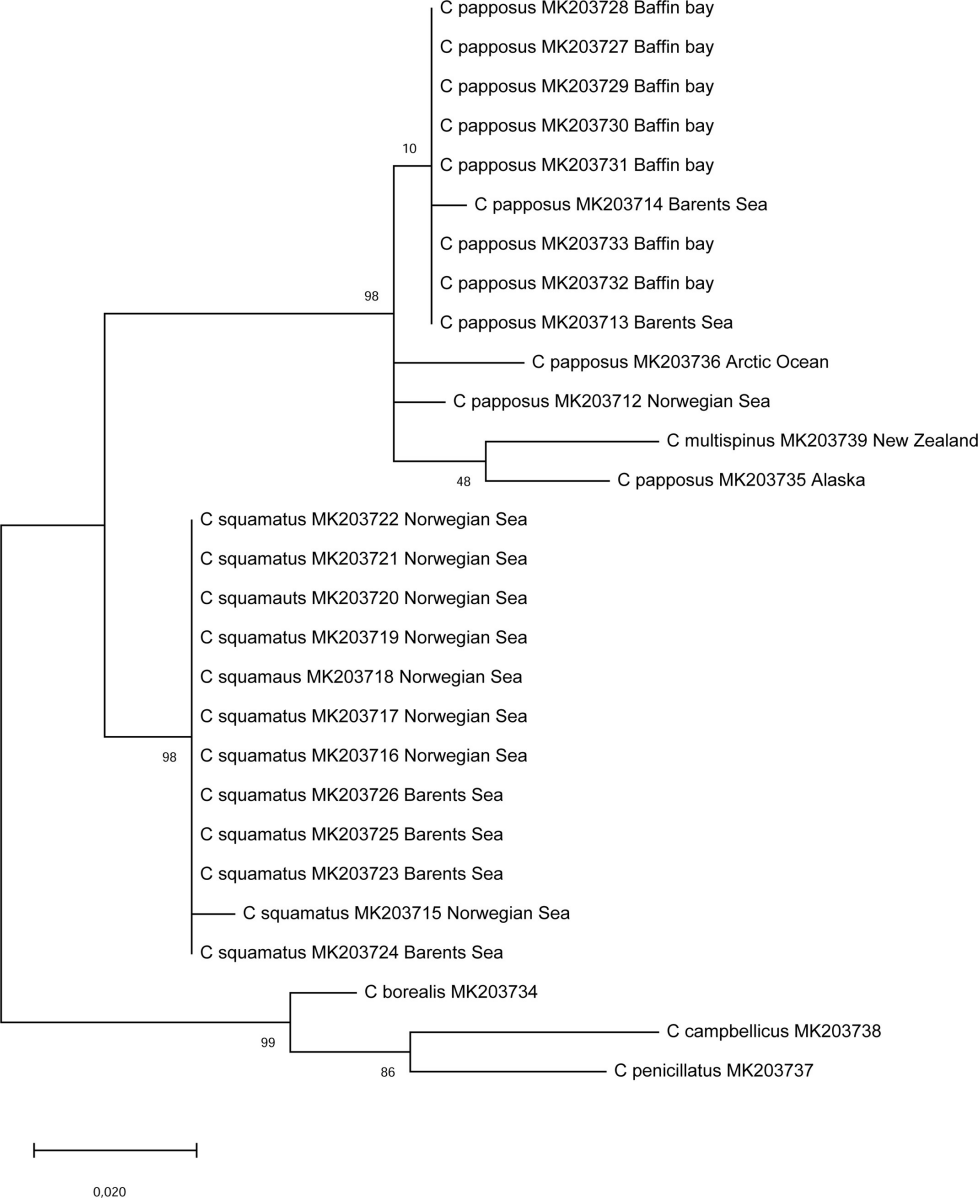
*Crossaster* relationships based on rDNA. Phylogenetic relationships among *C*. *papposus* and *C*. *squamatus* individuals, and representative *C*. *multispinus*, *C*. *borealis*, *C*. *penicillatus*, and *C*. *campbellicus*, was inferred from nuclear rDNA sequences using the Maximum Likelihood method. The tree with the highest log likelihood (-12678,68) is shown. The percentage of bootstrap replicates in which the associated taxa or individuals clustered together is shown next to the branches. The tree is unrooted and drawn to scale, with branch lengths measured by the number of substitutions per site.

The phylogenetic reconstruction based on nuclear rDNA sequences recovered *C*. *papposus* and *C*. *squamatus* as clearly separate taxa, in line with the evidence based on mitochondrial gene sequences. Again, *C*. *multispinus* clustered closely with *C*. *papposus*, but in contrast to the results of the mitochondrial gene based analyses, *C*. *multispinus* grouped among the *C*. *papposus* specimens rather than branching off as a separate lineage. Interestingly, it clustered most closely with the *C*. *papposus* specimen from the Pacific Ocean, though at a low bootstrap value (Fig 10). The other main group among *Crossaster* species, consisting of *C*. *borealis*, *C*. *campbellicus*, and *C*. *penicillatus*, was retained in the rDNA based phylogeny, though with a different internal branching order.

We found 3 *COI* haplotypes among 13 *C*. *squamatus* individuals, compared to 13 haplotypes among 23 *C*. *papposus* individuals, and nucleotide diversities of 0.15% and 0.78%, respectively. The amphioceanic *C*. *papposus* had similar nucleotide diversities in the Atlantic (0.29%, N = 13) and the Pacific (0.30%, N = 8), and a higher nucleotide diversity (0.57%, N = 5) than *C*. *squamatus* in their common distributional area in the NE Atlantic. The net number of nucleotide substitutions per site between *C*. *papposus* from the Pacific and the Atlantic/Arctic was 0.91%. The alignment of rDNA sequences for *Crossaster* species contained a total of 537 nucleotide positions and arrived at 413 nucleotide positions excluding gaps. Considering the 413 positions only, there were 4 genotypes among *C*. *papposus*, while a single genotype only was observed for *C*. *squamatus*. There were 15 fixed nucleotide differences between *C*. *papposus* and *C*. *squamatus*. Overall, the number of net nucleotide substitutions per site between the two putative species was 0.063 and 0.039 for *COI* and rDNA, respectively.

All of the *Crossaster* DNA sequences recovered by the present study are referred to in Table 1. Sequences were deposited in GenBank with the accession numbers KX451838-KX451847; MK270376-MK270394; MK203712-MK203739.

## Discussion

### Species delineation and phylogeny

Among the species currently assigned to the genus *Crossaster*, three species were originally described in the Atlantic: *C*. *papposus*, *C*. *helianthus* and *C*. *penicillatus*. As *C*. *penicillatus* was found in the SE Atlantic and Southern Ocean only, and the only record of *C*. *helianthus* to date is that of the holotype from Georges Bank in 1880, it would seem that *C*. *papposus* is the predominant representative of the genus in the North Atlantic, and the only one in the NE Atlantic. On the other hand, observations made by several authors suggested that variation could be contained within the *C*. *papposus* clade itself. Sladen [38] described the variety *C*. *papposus* var. *septentrionalis*, but based on a single specimen only, recorded from the Faroe Channel (-0,5⁰C). In 1900, Döderlein described individuals from Eggakanten in northern Norway, differing slightly from *C*. *papposus* in external morphology, which he tentatively termed *Solaster papposus* var. *squamata* (later *Crossaster squamatus* (Döderlein, 1900)). Subsequent researchers have held differing opinions as to whether the *papposus* and *squamatus* varieties should be considered valid species [11, 12, 13, 14, 39] or rather morphotypes associated with temperate (*C*. *papposus*) and colder (*C*. *squamatus*) waters [13, 14]. *C*. *squamatus* was maintained as a valid taxon in the North Atlantic Ocean (Rockall Trough) by A. M. Clark in Gage et al. [40] and Clark [41], but in Clark and Downey’s [5] compilation “Starfishes of the Atlantic”, it is omitted. The reason it was not included in this book could be that the authors considered it primarily as an Arctic species and, given the geographic constraint of the book, would avoid including species that did not occur in the Atlantic. *C*. *squamatus* is currently listed as an accepted species by WoRMS [42].

Here, we examined and unambiguously classified *Crossaster* specimens from the NE Atlantic as either *C*. *squamatus* or *C*. *papposus* based on external characteristics. Correspondingly, molecular markers representing both the mitochondrial and nuclear genomes clearly demonstrated that the two belong to separate lineages. The mitochondrial *COI* showed the typical level of divergence to be expected from a between species comparison. This is in agreement with the findings of Ward et al. [18] that the intraspecific divergence of echinoderm species ranged from 0 to about 3% with a mean of 0.62%, while congeneric divergence averaged 15.33%, based on *COI* sequences.

Nucleotide sequences of the 18S and 28S rDNA genes are traditionally utilized for phylogenetic inference and are able to resolve distant relationships due to their highly conserved primary sequence across metazoans. For the same reason, however, they are less informative for phylogenetic inference of closely related species. The variable ITS sequences contained within rDNA gene arrays have been less extensively used for phylogenetic inference, but the phylogenetic information content we were able to extract from ITS1 proved useful for investigating the closer relationships within the genus *Crossaster*. For *C*. *papposus* and *C*. *squamatus*, the present results based on the nuclear rDNA markers are in line with those based on mitochondrial sequence data, exhibiting some intraspecific variation and a clear phylogenetic resolution of the two putative species.

We analyzed six out of ten currently accepted *Crossaster* species worldwide, and recovered two major clades. The analysis suggests that *C*. *papposus* and *C*. *squamatus* belong to the same clade, as expected, while *C*. *papposus* and *C*. *multispinus* is the more closely related, possibly sister species. *Heterozonias alternatus*, *C*. *penicillatus*, *C*. *borealis*, and *C*. *campbellicus* constitute a robust group, which suggest that taxonomic classification within the same genus may be warranted. According to this analysis, neither *Crossaster* nor *Solaster*, as currently classified, constitutes natural clades. Instead, it suggests that *H*. *alternatus*, *C*. *penicillatus*, *C*. *borealis*, and *C*. *campbellicus* constitute a group nested among *Solaster* species, while the placement of *Solaster* species in the phylogeny remains uncertain due to low bootstrap support. A more comprehensive sampling of the genus, including the Pacific *C*. *scotophilus* (Fisher, 1913), *C*. *japonicus*, and *C*. *diamesus* (Djakonov, 1932), as well as other solasterid species, and a multigene approach, will be needed to further resolve the phylogenetic relationships of *Crossaster* species.

So far, molecular data on *Crossaster* species are scarce. A previous study [43] based on partial sequences of two mitochondrial rDNA genes (12S and 16S) and one nuclear protein coding gene (early stage histone H3) failed to recover the close relationship between *C*. *papposus* and *C*. *multispinus* that we identified in the present study. This previous study, however, was aimed at resolving higher order relationships among asteroids rather than the finer twigs of the phylogenetic tree. Indeed, upon reanalysis of the available 12S, 16S and histone H3 sequence data of *Crossaster* species only (*C*. *papposus*, *C*. *multispinus* and *C*. *borealis*), and using *Lophaster furcilliger* as the outgroup, we found a close relationship between *C*. *papposus* and *C*. *multispinus*, and a branching order of the species included in perfect agreement with the results of the current study.

*C*. *multispinus* has been recorded from the South Pacific Ocean only, in specific from South and Southeast Australia (Gabio Island and Disaster Bay), Tasmania, Macquarie Island and New Zealand [30, 44, www.iobis.org]. Thus, it is evident from the currently available data that the widely distributed amphioceanic *C*. *papposus*, has at least one closely related representative, *C*. *multispinus* and *C*. *squamatus*, in each of the Pacific and Atlantic Oceans. It would be interesting to further investigate the population histories and differentiation of the closely related *C*. *papposus*, *C*. *multispinus* and *C*. *squamatus*, and to identify their adaptative genetic variation, using a population genomic approach.

### Biogeography of *C*. *papposus* and *C*. *squamatus*

Although the current sampling of *C*. *papposus* and *C*. *squamatus* remains limited both in terms of geographic extent and the number of individuals, it seems evident that the two species differ widely in their distribution, as well as their population genetic parameters. *C*. *papposus* is genetically more diverse than *C*. *squamatus* in terms of the number of haplotypes and nucleotide diversities. The molecular phylogenies showed evidence of geographic structure for *C*. *papposus* in that specimens from the Pacific Ocean clustered separately with strong bootstrap support and specimens from Baffin Bay/Greenland clustered tightly with those from the Barents Sea. Also, the branching patterns of specimens from the Arctic and Norwegian Sea are compatible with geographic structuring, but further sampling of individuals would be required to establish a proper phylogeography of the species.

Our estimate of 0.91% sequence divergence of *COI* between trans-Arctic *C*. *papposus* is in line with other recent estimates, 1.24% and 1.03%, obtained by [45] and [46], respectively. The level of divergence is relatively low compared to several other trans-Arctic sister clades and suggests a recent separation dating back some 3–400 000 years with a divergence rate of 2.8%/million years [46]. The observation made by Loeza-Quintana & Adamowicz [46] that trans-Arctic interchange seems to be favoured by taxa that have shallow versus those that have deep water distributions is in line with the depth distributions of *C*. *papposus* and *C*. *squamatus*. Thus, *C*. *papposus* would seem to be a highly abundant and widely distributed species, with a higher potential for dispersal, but able to adapt locally and diversify, which could entail incipient speciations. In contrast, *C*. *squamatus* seems to lack in numbers and genetic diversity, maybe due to more restricted habitats, lack of dispersal capabilities, special adaptations, and a competitive disadvantage compared to *C*. *papposus*. Also, we note that branch lengths of the molecular phylogenies suggest that *C*. *papposus* experiences higher molecular evolutionary rates than those of its close relatives, *C*. *squamatus* and *C*. *multispinus*. Higher rates in *C*. *papposus* could be related to shorter generation times, which in turn could be due to higher temperatures and concomitant increase in developmental rates, but we are not aware of any data on generation times in *Crossaster* species so far, to support or contradict such a speculation.

Based on the specimens analysed here, we found a consistent morphological differentiation between *C*. *papposus* and *C*. *squamatus* and a corresponding genetic differentiation, with no evidence of introgression between the two. We did identify a few specimens, however, with a combination of dense dorsal structures (as for *C*. *squamatus*) and several papulae within each membranaceous space (as for *C*. *papposus*), generally with 5–7 and occasionally 3–5 furrow spines, during our previous examination of *Crossaster* specimens from the three marine surveillance programs mentioned (BIOFAR, BIOICE and MAREANO). Such a combination of morphological characteristics from both taxa is suggestive of hybridization. Further molecular analyses, targeting both mitochondrial and nuclear genes, would be required to resolve the issue, but the specimens currently available are preserved in formalin and therefore less appropriate for DNA analyses.

The geographic distribution of *Crossaster papposus* and *C*. *squamatus* overlap. The distribution of *Crossaster papposus* in the Atlantic Ocean is south along the east coast of north America, from Newfoundland and Labrador to about 40°N; Spitsbergen, north to Nordaustlandet (Barents Sea); in the NE Atlantic from Scandinavia (Finnmark, including Tromsøflaket, and south all along the Norwegian coast) to the southern North Sea, all around the British Isles, Iceland south to northern Brittany, all around the Faroe Islands on the shelf [5, 11, 22, 47–50, Ringvold et al. In prep.; this study]. According to records from CASIZ database it also occurs at the strait of Gibraltar in Spain. *C*. *papposus* is also widely distributed in the North Pacific [51].

*C*. *squamatus* has been recorded in Norwegian waters from Finnmark and Eggakanten (Fig 1), south to the border of Nordland and Trøndelag Counties (65°N) [12, this study], by Hansson [52] stated as “NW-NE Finnmark, slope of Norway S., south to 60° N”. It is also distributed all around the Faroe Islands, including the Faroe Channel; Iceland; north to Nordaustlandet in the archipelago of Svalbard; western Barents Sea; east and west of Greenland and south to the Hebridean slope (56°N) [11, 14, 22, 29, 40, 49, 53–59, Ringvold et al. in prep.]. It has also been recorded from the NW Atlantic, Newfoundland, Baffin Bay and Smith Sound [48, 60]. Worldwide, *C*. *papposus* is recorded from 0–1200 m depth [5, 11], and *C*. *squamatus* from cold water areas, from 100–1600 m depth [11, 50]. However, since previous opinions have differed with respect to taxonomic assignment of *Crossaster* found in the NE Atlantic, and misidentifications might have occurred, our understanding of the geographic distribution, as well as the depth distribution, might be subject to change in the future.

While there is a distributional overlap of *C*. *papposus* and *C*. *squamatus*, the abundance-weighted mean depth is shallower for *C*. *papposus* than for *C*. *squamatus*, based on morphologically identified, mainly formalin preserved materials, from the Faroe Islands (BIOFAR), Iceland (BIOICE), and Norway (MAREANO). *C*. *papposus* was found on the shelf in temperate water masses, whereas *C*. *squamatus* showed abundance-weighted mean depth below the shelf break (below 500 m depth), in the transition zone with mixed, colder water masses, including negative temperatures [22, Ringvold et al. In prep.] (Fig 7). At a few MAREANO stations, specimens were identified as *C*. cf. *papposus*. If omitting these questionable specimens, the average depth for *C*. *papposus* would have been even shallower, as observed in BIOFAR and BIOICE data. Zoogeographical analysis in Einarsson [61] rests mainly on several of Th. Mortensens publications (e.g. Mortensen [11]), supporting our findings, in placing *C*. *papposus* as part of the arctic-boreal fauna, and *C*. *squamatus* as part of the Arctic deep basin fauna.

**Fig 11:**
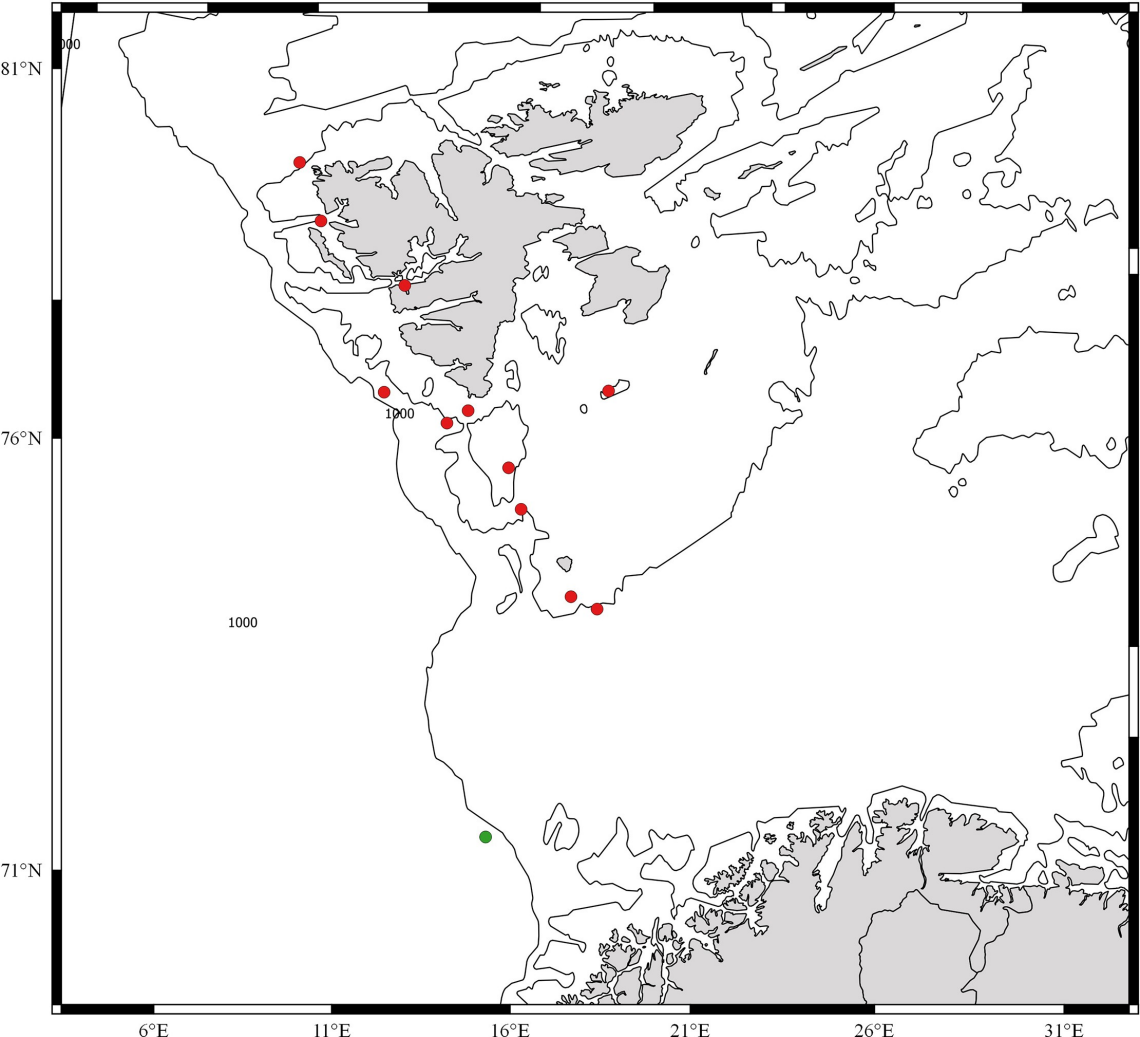
**Recordings of *Crossaster papposus* (red dots) and *C*. *squamatus* (green dots), the former species sampled by the Olga expedition, and the latter from the North-Sea Expedition [12].** *C*. *papposus* is distributed close to the Svalbard shore, whereas the one recording of *C*. *squamatus* was at the shelf-break.

Correspondingly, Döderlein’s [12] recordings of *C*. *papposus* from 11 stations (mainly Olga expedition) and the holotype of *C*. *squamatus* (North-Sea Expedition, st. 200) show that *C*. *papposus* at Svalbard is also distributed close to the shore (36–200 m depth), whereas the one recording of *C*. *squamatus* was at 1134 m depth, at -1°C (Fig 11). Jones et al. [62] recorded only *C*. *squamatus* (by uv-photo and video images) in the deep Faroe Channel, at stations ranging from ~ 1000 m to 1200 m depth. The bottom waters in the channel at depths below 800 m is ~ -1° C [63].

Several features, such as distribution, shape of calcareous ossicles, and genetic differentiation, have been associated with temperature for several other species as well, and studies have related the distribution of macro-invertebrates to water mass as defined by both temperature and salinity [64–66]. Previous studies from the Norwegian Sea have shown that the transition zone, an area with mixture of water masses, represents maximum species diversity, and a major shift in benthic species composition (e.g. [20, 67, 68]). Temperature alone is also an important abiotic factor regarding distribution of benthic species, including Asteroidea (e.g. [69–71]). Important faunal boundaries, found globally, are believed to occur around the shelf/slope break at 200–500 m, and around 1000–1400 m depth (e.g. [20, 67, 72]). Gage [71] found a comparative echinoderm faunal boundary at 800–1000 m in the Rockall Trough, and Howell et al. [67] asteroid faunal boundaries at Porcupine Seabight at 110 m, ~700 m, and 1700 m. In both studies the boundaries were related to both depth of the thermocline and water mass structure.

A study of the deep-water amphipod *Eurythenes gryllus* (Lichtenstein in Mandt, 1822) suggests that depth (or pressure), together with topography, is a significant driver in allopatric (= geographic) speciation where populations become separated and isolated over a long period, and interfering with genetic interchange due to e.g. different selective pressures or mutations of the different populations. Three distinct morphological forms of the species have been detected, varying in terms of pereonites and pleonites, the shape of coxa 2, and the first and second gnathopods [73]. Temperature has been discussed as a controlling ecological factor in the deep sea [74–76], also to *E*. *gryllus*, and may lead to genetic differentiation and speciation [77]. This conclusion is reasonable when comparing bathyal to abyssal populations due to distinct bottom temperatures, but not regarding populations in the abyssal and hadal trenches with more similar temperatures [73]. The distribution of *Crossaster papposus* and *C*. *squamatus* overlap to some extent, but they seem to prefer different depth zones with different temperatures, hence both abiotic factors (temperature and depth) may have contributed to the differentiation of the two.

Temperature is also suggested to cause changes in the calcareous skeleton/plates in e.g. Bryozoa. This is seen in the species complex *Watersipora* spp., generating phylogroup-specific fragments. Warm water colonies show irregular, multilobed morphology compared to cold-water colonies, which are more regular and circular in shape [78]. The same was observed in the asteroid genus *Bathybiaster* where the dorsal plates of the warm water form are described as star-shaped and overlap, whereas the cold water form shows round plates which do not overlap. Grieg [79] therefore suggested a warm- and cold-water species within the asteroid genus *Bathybiaster*, namely *B*. *vexillifer* (W. Thomson, 1873) and *B*. *robustus* (Verrill, 1894). The two species were synonymized by Koehler [80], and followed by Mortensen [11] and Fisher [81], but maintained by Clark [82]. Today, *B*. *robustus* is synonymized with *B*. *vexillifer* (www.marinespecies.org). Similarly, it has been suggested that the morphological differentiation between *C*. *papposus* and *C*. *squamatus* might be caused by temperature [11–14, 40, 83], but the genetic distances and phylogeny revealed by the present study makes it evident that they belong to clearly divergent lineages, consistent with species status.

While Ocean Acidification (OA) is generally considered a major threat to marine ecosystems, lowered pH was shown to increase growth of *C*. *papposus* developmental stages [84]. In contrast, one of its main prey species, *Asterias rubens* Linnaeus, 1758, which has also been shown to be more prone to diseases during OA due to immune suppression, will be negatively impacted [85]. At the Faroe Islands, *C*. *papposus* and its prey *A*. *rubens*, are found mainly on the shelf, in relatively warm water and strong currents (40–90 cm s^-1^) [30], as supported by Gale et al. [86] in finding a maximum density depth for both species between 0 and 100 m in a study from Atlantic Canada. In the same area, *C*. *squamatus* occurs mostly at deeper and colder waters with weaker currents (12–41 cm s^-1^), indicating it may have different food preferences. Bearing these differences in mind, it should be interesting to explore niche separation between the species, and whether they are differentially affected by OA and other human induced environmental changes. Changes in the population sizes of these species, which are both top predators, could have important consequences for community structure and functioning.
