# Correction: On the genus *Crossaster* (Echinodermata: Asteroidea) and its distribution

**DOI:** 10.1371/journal.pone.0229318

**Published:** 2020-02-12

**Authors:** Halldis Ringvold, Truls Moum

A colon is incorrectly placed in the species name in the article title. The correct title is: On the genus *Crossaster* (Echinodermata, Asteroidea) and its distribution. The correct citation is: Ringvold H, Moum T (2020) On the genus *Crossaster* (Echinodermata, Asteroidea) and its distribution. PLoS ONE 15(1): e0227223. https://doi.org/10.1371/journal.pone.0227223

In the “Morphology” subsection of the Results, there is an error in the first sentence of the third paragraph. The correct sentence is: In *C*. *squamatus*, the dorsal skeleton is scale-like, formed by irregularly shaped plates with little or no membranaceous spaces, as seen in the MAREANO specimens (Fig 4), and with only singular papula. There is also an error in the first sentence of the fourth paragraph of this subsection. The correct sentence is: The dorsal skeleton of the borrowed *C*. *multispinus* (Fig 5) consists of narrow bars forming an irregular reticulum of plates with large membranaceous spaces, as in *C*. *papposus*, and also described by Clark [30].

**Fig 4:**
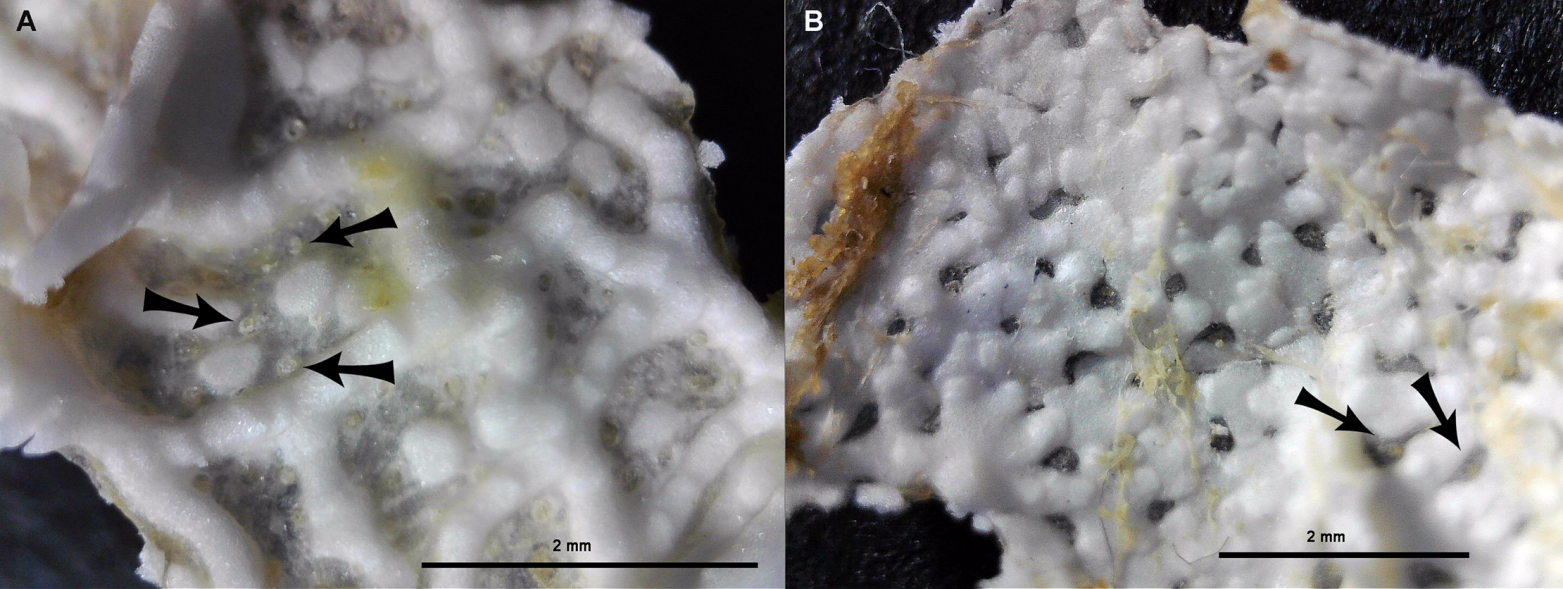
Dorsal skeleton of *Crossaster papposus* and *C*. *squamatus*. a (left). The dorsal skeleton of *C*. *papposus* is formed by narrow bars with large membranaceous spaces. Specimen recorded at MAREANO station 1218–471 (R = 2 cm), and b. The dorsal skeleton of *Crossaster squamatus* is scale-like, with irregular shaped plates, and with little membranaceous space. Specimen recorded at MAREANO station 1086–438 (R = 1,8 cm). The arrows show papulae within membranaecous space. The dorsal skeleton samples are cut out and photographed from below. (Photo credit: Halldis Ringvold/ Sea Snack Norway.).

**Fig 5:**
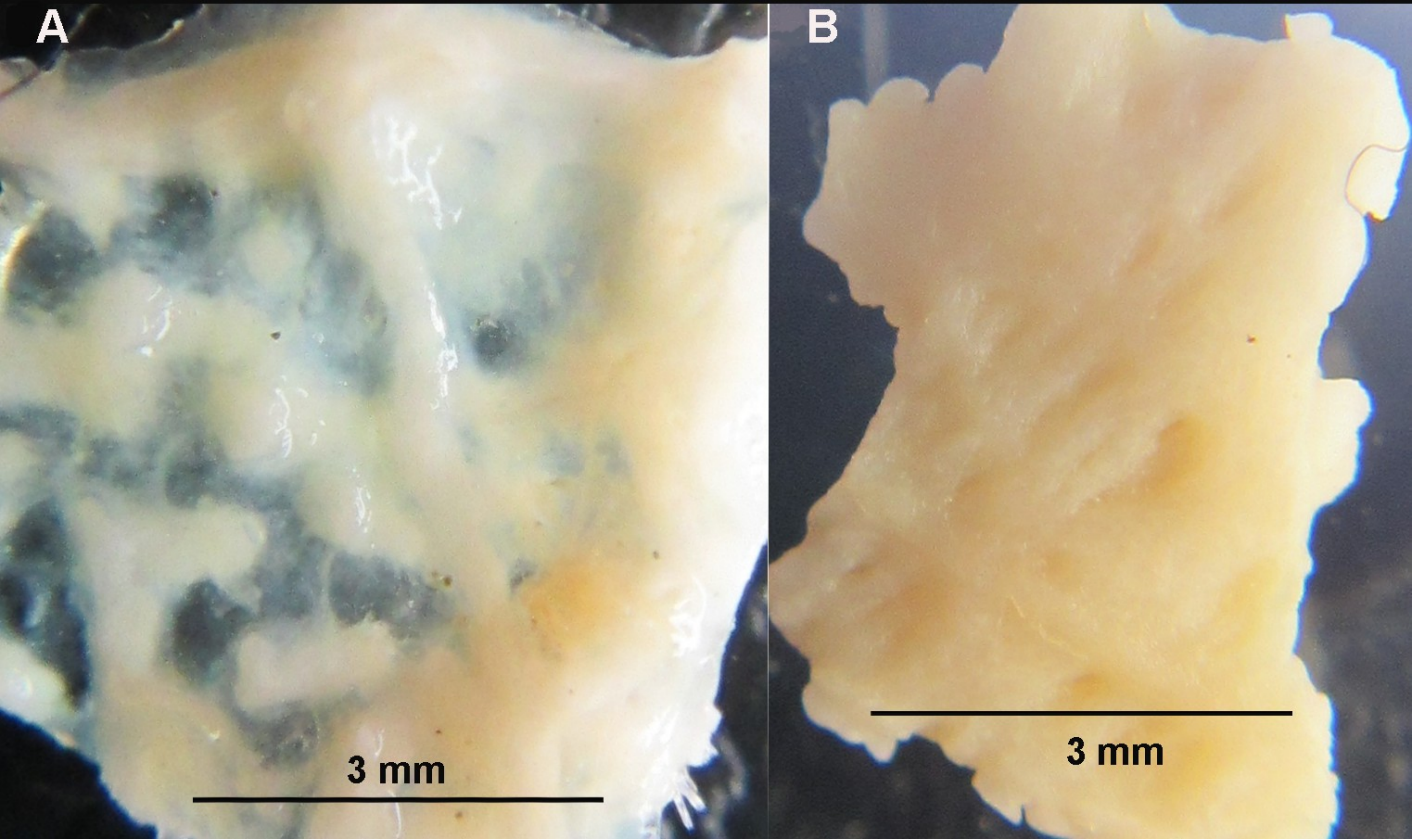
Dorsal skeleton of *Crossaster multispinus* and *C*. *campbellicus*. a (left). Dorsal skeleton of *C*. *multispinus* is, like for *C*. *papposus*, formed by narrow bars with large membranaceous spaces, and b. In *C*. *campbellicus* the dorsal skeleton is more compact.

The captions for Fig 4 and Fig 5 are incorrect. Please see the complete, correct Fig 4 and Fig 5 captions here.
